# Transatlantic Delphi Consensus on the Common Iliac Artery Sealing Zone in Endovascular Aorto-Iliac Aneurysm Repair (the DECIDE Study)

**DOI:** 10.1177/15266028241295919

**Published:** 2024-11-20

**Authors:** Maria-Annette Kooijman, Mario D’Oria, Luca Bertoglio, Isabelle Van Herzeele, Ross Milner, Jean-Paul P.M. de Vries, Richte C.L. Schuurmann

**Affiliations:** 1 Division of Vascular Surgery, Department of Surgery, University Medical Center Groningen, Groningen, The Netherlands; 2 Division of Vascular and Endovascular Surgery, Department of Medical Surgical and Health Sciences, University of Trieste, Trieste, Italy; 3 Division of Vascular Surgery, University of Brescia, ASST Spedali Civili Hospital of Brescia, Brescia, Italy; 4 Department of Thoracic and Vascular Surgery, Ghent University Hospital, Ghent, Belgium; 5 Division of Vascular Surgery and Endovascular Therapy, University of Chicago Medicine, Chicago, IL, USA

**Keywords:** abdominal aortic aneurysm, endovascular aneurysm repair, Delphi consensus, stents, iliac artery, guidelines

## Abstract

**Objective:**

Knowledge of hostile factors and their influence on long-term seal in the iliac landing zone is limited. Currently endorsed clinical practice guidelines lack structural evidence on how the iliac landing zone should be assessed in the pre-, intra-, and postoperative phases. The goal of this study was to obtain an international, expert-based consensus on the definition of a hostile iliac landing zone, on how to size and plan stent-grafts to optimize sustainable distal seal, and on the postprocedural follow-up protocol.

**Methods:**

Delphi consensus methodology was used, involving a panel of international vascular surgeons experienced in endovascular aneurysm repair (EVAR). The first round consisted of open-ended and multiple-choice questions to explore current practices, with subsequent rounds refining statements through a 4-point Likert scale. Consensus was defined as >75% agreement or disagreement, and the analysis included stability testing and strength of consensus.

**Results:**

The study engaged 77 international vascular surgeons, reflecting diverse geographic locations and hospital affiliations. Consensus was achieved on critical preoperative planning elements for EVAR, including a clear definition for a hostile iliac landing zone. The importance of computed tomography angiography for postoperative follow-up imaging was emphasized, including evaluating distal seal length and recommending specific timing for follow-up computed tomography scans and intervention strategies for diminishing iliac seal.

**Conclusions:**

This international expert-based Delphi consensus establishes a comprehensive set of consensus-driven recommendations focused on the definition and management of hostile iliac landing zones in EVAR. The key recommendation of this study is the definition of a hostile iliac landing zone as short (<15 mm), wide (>24 mm), or conical (>10% diameter difference along the landing zone). Although consensus was achieved on several critical aspects, the study also reveals ongoing debates and considerations that warrant further exploration, including how to tackle diminishing seal without a type IB endoleak.

**Clinical Impact:**

This Delphi consensus introduces a standardized definition of a hostile iliac landing zone as short (<15 mm), wide (>24 mm), or conical (>10% diameter difference), clinicians now have a clearer framework for assessing complex anatomies. This study provides a comprehensive set of consensus-driven recommendations focused on the definition and management of hostile iliac landing zones in EVAR which gives guidance where current guidelines lack specificity, particularly for distal iliac sealing. The study also reveals ongoing debates and considerations that warrant further exploration, including how to tackle diminishing seal without a type IB endoleak.

## Introduction

Endovascular aneurysm repair (EVAR) has emerged as the preferred therapeutic option for treating abdominal aortic aneurysms, especially in high-risk patients.^1^ The Achilles’ heel of EVAR is the occurrence of type IA and IB endoleaks with the subsequent need for lifelong follow-up and reinterventions.^2^ Durability of EVAR remains a concern, especially in patients with a longer life expectancy. Also, cost-effectiveness of EVAR is greatly impacted by the need for secondary interventions. Sustainable seal of the main body and limbs is crucial to obtaining a durable result.

Challenging anatomy of the infrarenal aortic neck has been addressed in numerous studies (including a Delphi consensus), specifically focusing on the concept of a “hostile neck.”^3–6^ These studies highlight the importance of accurate sizing and planning, but mostly focus on the infrarenal neck. The criteria for the proximal landing zone are also well defined in the instructions for use of commercially available endografts. For the distal (iliac) landing zone, however, there is less knowledge of hostile factors and their influence on long-term seal.^2^ Currently endorsed clinical practice guidelines lack structural evidence on how the iliac landing zone should be assessed in the pre-, intra-, and postoperative phases.^7–9^

The goal of this study was to obtain an international, expert-based consensus on the definition of a hostile iliac landing zone, on how to size and plan stent-grafts to optimize sustainable distal seal, and on the postprocedural follow-up protocol.

## Materials and Methods

### Study Design

The Delphi consensus methodology, which was used in this study, is a recognized and well-established approach for attaining consensus among a panel of experts on subjects lacking empirical evidence.^10^ In this study, a panel received a series of statements on the landing zone of the common iliac artery, and feedback from the participants was incorporated through several rounds until consensus was reached.

Before the panel was selected, a steering committee was assembled comprising clinical researchers and vascular surgeons with extensive experience in EVAR and Delphi consensus. The steering committee was responsible for the design of the study, including selection of panelists, formulation of the questions and statements and their reformulation throughout the rounds, as well as being responsible for the statistical analysis, interpretation of the results, and composition of this report.

The first round of this Delphi consensus consisted of open-ended and multiple-choice questions aiming to investigate the current practices and preferences of the panelists. These questions were carefully selected by the steering committee based on knowledge deficits in the current guidelines and available literature.^7,9^ Panel members had the opportunity to suggest additional statements and to comment on all questions in the first round. The steering committee then formulated a list of statements with a 4-point Likert scale (fully agree, agree, disagree, fully disagree) for the second round.

Agreement (fully agree, agree) of ≥75% of the panelists was considered a consensus to agree, and disagreement (disagree, fully disagree) of ≥75% of the panelists was considered negative consensus. Panelists who disagreed with a statement were asked to elaborate why they disagreed. Statements that did not reach 75% consensus were evaluated, including the reasons for disagreement. The steering committee rephrased these statements to aim for consensus in the third round. If consensus was not probable at the end of the second round, the statement was withdrawn. Consensus was set as a closing criterion for the statements. All statements from the second round that reached consensus were repeated in the third round to test for group stability. Consensus strength was graded based on the percentage of full and overall agreement (Table 1).

**Table 1.** Strength Grading Definitions Used in the Development and Analysis of Consensus.

Grade	Description	Definition
**A**	Very strong	Fully agree ≥ 75%
**B**	Strong	Fully agree < 75% Overall agreement ≥ 80% Fully disagree < 5%
**C**	Fair	Fully agree < 75% Overall agreement ≥ 80% Fully disagree ≥ 5%
**D**	Poor	Fully disagree ≥ 10%

### Expert Panel

The steering committee identified a panel of international vascular surgeons experienced in the field of EVAR. These panel members were selected either based on previous publications in the field or from suggestions from the steering committee. An attempt was made to gather a heterogeneous group of vascular surgeons with different transatlantic nationalities who were practicing in academic and teaching hospitals. Experts who did not respond to a round were excluded from participating in subsequent rounds. The steering committee members were not part of the expert panel.

### Data Collection

The Delphi consensus was conducted via online surveys, which were completed and collected using REDCap (Vanderbilt University, Nashville, TN, USA). Nonresponding panelists received reminder emails after 7 and 14 days subsequently. Identity of the participants was only known to the steering committee, and all responses were analyzed in aggregate in compliance with privacy laws. Panelists could withdraw from the Delphi procedure at any time. Institutional Review Board Approval was not required because no patient data were processed for this study. The study complied with European privacy legislation.

### Stability Testing

Statistical analysis for stability testing was conducted using SPSS 28.0 statistical software (IBM, Armonk, NY, USA). Group stability was primarily tested using Pearson χ2 testing to conform to the methods of Dajani et al.^11^ After this, cross tabs were used to identify the percentage of overlap between agree versus do not agree the respondents’ answers in rounds two and three to support our group stability values. *p* values were considered statistically significant with an α of <.05.

## Results

### Expert Panel

The steering committee invited 127 experts to participate in round 1, of whom 82 (65%) agreed to participate and responded to the first questionnaire. Of these 82 participants, 80 (98%) responded to the second round, and 77 (94%) to the third round, and their responses were included in the final analysis. Fifty-four panelists were based in Europe, 19 in Northern America, and four in Asia and New Zealand, representing 17 different countries. Of the 77 panelists, 81% were working in an academic center and 16% in a teaching hospital. Most of the panelists worked in a center where >100 elective procedures may take place annually, and 10 to 30 urgent EVARs are performed annually. The panelists were a mean age of 50.8 ± 8.3 years, and 12% were women (Table 2).

**Table 2.** Characteristics of Participating Panelists in the DECIDE Study (n=77).

Characteristic	Data value
*Sex*	
Female	12
Male	87
NA	1
*Age (mean±SD), year*	50.8±8.3
*Continent*	
Europe	70
North America	25
Other	5
*Type of hospital*	
Academic:	81
Public:	16
Other	1
*Elective cases in panelist’s center per year*	
<50	4
51 –100	39
101–150	25
151–200	10
>200	22
*Urgent cases in panelist’s center per year*	
<10	12
10–30	70
>30	17

Values are given in percentage unless stated otherwise.

### Selection of Statements

The first round consisted of 25 questions about current clinical practice. From this, 37 statements were constructed with an aim for consensus with a 4-point Likert scale, and submitted in the second round (Tables 3–5). After the second round, three statements were modified and resubmitted in the third round, two statements were withdrawn, and 32 statements were resubmitted without modification (Supplementary Table S1).

**Table 3.** Statements in the Delphi Consensus Concerning the Planning and Preoperative Evaluation of the Iliac Landing Zone in Endovascular Aneurysm Repair.

Statement	Consensus round 2	Consensus round 3	Consensus strength round 3	*P*-value Pearson χ^2^	Overlap round 2 vs 3
1. The most appropriate method for sizing and planning an elective EVAR procedure should be based on a computed tomography angiography and by using dedicated post processing software analysis	97	99	A	.238	98.7
2. The IFU should be followed in elective EVAR to determine the distal landing zone	96	96	B	.213	97.4
3. The length of the distal landing zone should always be measured preoperatively	96.1	100	A	.238	96.0
4. The length needed for proper sealing in the distal landing zone should ideally be at least 2 cm and never less than 1.5 cm	93	93	B	.213	92.0
5. The sizing of the endograft limb diameter is based on the maximum diameter at the anticipated distal landing zone	97	100	B	.213	97.3
6. Round 2: The maximum diameter of the distal landing zone should ideally be 24 mm Round 3: The maximum diameter of the distal landing zone should ideally be 22 mm	70.1	92*	B	.213	70.0*
7. Tortuosity of the distal landing zone should be taken into account when planning an EVAR	100	100	B	.092	100
8. Calcification and thrombus of the distal landing zone should be taken into account when planning an EVAR	99	100	B	.238	98.7
9. The distal landing zone should be free of circumferential thrombus/calcification	77	84	B	.213	81.8
10. Conicity of the distal landing zone should be taken into account when planning an EVAR	100	100	B	.092	100
11. A hostile iliac landing zone can be defined as any of the following characteristics: short (<15 mm), wide (>24 mm), or conic landing zone (>10% diameter difference along the landing zone)	92	93	B	.213	90.1
12. The distal landing zone is defined as a straight segment without >10% change in diameter	95	96.	B	.213	93.2
13. Round 2: The iliac anatomy determines the chosen EVAR platform (model/type) Round 3: The aortic anatomy rather than the iliac anatomy determines the chosen EVAR platform (model/type)	71	49*	D*	.213	52*
14. At least one internal iliac artery should be preserved, if possible preserving both internal iliac arteries should be considered	100	96	A	.238	96.0
15. If it is necessary to preserve the internal iliac artery, bilateral iliac branch graft should be placed rather than one device and occlusion of the contralateral internal iliac artery	68	**	**	**	**
16. Even if the length of the distal landing zone is >2 cm, the entire common iliac artery should be covered to the origin of the internal iliac artery	87	84	B	.213	97.3
17. When an iliac branched graft is used, the iliac anatomy will determine the branch platform (model/type)	91	97	B	.213	88.0

Consensus values and overlap between rounds are presented in percentages.

* Statement reformulated after the second round; **Statement dropped after the second round.

**Table 4.** Statements in the Delphi Consensus Concerning the Intra-Operative Management of the Iliac Landing Zone in Endovascular Aneurysm Repair.

Statement	Consensus round 2	Consensus round 3	Consensus strength round 3	*p*-value Pearson χ^2^	Overlap round 2 vs 3
18. To assess the distal seal at the end of the EVAR procedure, angiography is the preferred imaging modality	96	97	B	.213	93.5
19. An angiogram is sufficient to judge the significance of a type IB endoleak during the procedure	88	94	B	.213	89.6
20. If there is a *high-flow* type IB endoleak present perioperatively, the best treatment is to intervene directly during the index procedure without occluding the internal iliac artery	90	95	B	.213	89
21. If there is a *low-flow* type IB endoleak present perioperatively, the best treatment is to intervene directly during the index procedure without occluding the internal iliac artery	80	84	B	.213	85.5
22. If there is sufficient length, a perioperative type IB endoleak should be treated by extending the distal landing zone	95	100	B	.238	94.7
23. If there is insufficient length, a perioperative type IB endoleak should be treated by molding the distal landing zone with a noncompliant balloon first	90	97	B	.213	89.3
24. If there is insufficient length, and molding is unsuccessful, a perioperative type IB endoleak should be treated by extending the distal landing zone, preferably using an iliac branched graft if anatomically feasible	82	84	B	.213	84.4
25. The bell bottom technique may be considered a high risk procedure for later development of type IB endoleaks	87	91	B	.238	88.2

Consensus values and overlap between rounds are presented in percentages.

**Table 5.** Statements in the Delphi Consensus Concerning the Postoperative Follow-Up of the Iliac Landing Zone in Endovascular Aneurysm Repair.

Statement	Consensus round 2	Consensus round 3	Consensus strength round 3	*p*-value Pearson χ^2^	Overlap round 2 vs 3
26. Round 2: The first postoperative CTA should be done within 30 days Round 3: The first postoperative CTA should be done within 2 months	74	84*	B	.213	86.8*
27. The achieved distal seal length should be measured on the first follow-up imaging study	90	91	B	.213	96.1
28. If the achieved distal seal is measured postoperatively, this should be based on a computed tomography angiography and by using dedicated post processing software	87	90	B	.213	94.8
29. The achieved distal seal length should ideally be at least 2 cm and never less than 1.5 cm	91	97	B	.213	93.5
30. If the achieved distal seal length is less than 1.5 cm without the presence of a type IB endoleak, watchful waiting is recommended	91	97	B	.213	90.8
31. The second postoperative imaging should be done within 1 year postoperatively using CTA	80	83	B	.213	89.3
32. The preferred follow-up imaging to determine distal landing zone is CTA rather than duplex ultrasound	88	92	B	.213	89.2
33. The achieved distal seal length should be measured on follow-up CTAs and compared with previous scans to detect changes	91	92	B	.213	93.5
34. Diminishing iliac seal without type IB endoleak during follow-up must lead to intensified follow-up	92	95	B	.213	92.2
35. Diminishing iliac seal without type IB endoleak during follow-up is reason for intervention	39	**	**	**	**
36. If the distal seal length on the second follow-up imaging is less than 1.5 cm without the presence of a type IB endoleak, watchful waiting is recommended	84	96	B	.213	90.4
37. If there is a type IB endoleak present on the follow-up imaging, it is recommended to extend with an additional stent graft. If there is no sufficient length an iliac branched graft should be considered if anatomically feasible	95	99	A	.213	93.5

Consensus values and overlap between rounds are presented in percentages.

* Statement reformulated after the second round; **Statement dropped after the second round.

### Planning and Preoperative Evaluation

There were 17 statements on preoperative planning (Table 3) in which experts agreed that sizing and planning elective EVAR procedures should be based on a computed tomography (CT) angiography (CTA) with dedicated postprocessing software. The experts agreed that instructions for use should be respected to determine the distal landing zone, emphasizing the importance of measuring length and basing endograft diameter on the maximum diameter at the anticipated distal landing zone. There was consensus on the ideal length and diameter; namely, that the distal landing zone should ideally be at least 20 mm and never <15 mm and that the diameter should ideally be <22 mm. Experts agreed on the importance of tortuosity, calcification, thrombus, and conicity when planning EVAR. In addition, the consensus highlighted the need to cover the entire common iliac artery to the origin of the internal iliac artery, with the choice of iliac branched graft determined by iliac anatomy.

The before mentioned statements resulted in an accepted definition for a hostile iliac landing zone: a hostile iliac landing zone can be defined as any distal landing zone with any of the following characteristics: short (<15 mm), wide (>24 mm), or conical (>10% diameter difference along the landing zone).

### Intraoperative Phase

All intraoperative statements are shown in Table 4. Completion angiography was the preferred imaging method to assess the distal seal at the end of an EVAR procedure and sufficient to judge the significance of a type IB endoleak during the procedure. The experts also agreed that the best treatment in both high-flow and low-flow type IB endoleaks is to intervene immediately during the index procedure. When sufficient length remains in the distal common iliac artery, an intraoperative IB endoleak should be treated by extending the distal landing zone, and when the length is insufficient, it should be treated by molding with a noncompliant balloon first. When the length is insufficient and molding is unsuccessful, an intraoperative type IB endoleak should be treated by extending the distal landing zone, preferably using an iliac branched graft if anatomically feasible. The bell-bottom technique is considered a high-risk procedure for later development of type IB endoleaks.

### Postoperative Phase

The full set of postoperative statements and outcomes are shown in Table 5. Key recommendations that reached consensus for imaging follow-up were that the first postoperative CTA should be done within two months after EVAR and should include measurement of the distal seal length. The distal seal between the endograft limb and iliac artery should be ideally at least 20 mm, but not <15 mm. The second postoperative CTA should be within 1 year, and the distal seal should be compared with previous scans to detect changes. If there is diminishing seal without endoleaks, intensified follow-up and imaging is recommended.

## Discussion

This Delphi consensus has established the novel definition of a hostile iliac landing zone as short (<15 mm), wide (>24 mm), and/or conical (>10% diameter difference along the landing zone). This provides a clear framework for identifying challenging anatomies and harmonizing reporting in future research.

Iliac distal landing zone problems, such as type IB endoleaks, have been vastly underreported in the scientific literature,^2,12^ even though type I endoleaks are the leading cause of late rupture after EVAR.^13,14^ Even though current international guidelines represent a commendable effort to summarize the sometimes limited literature, they acknowledge that some recommendations are based on low level evidence. When guidelines are derived from lower levels of evidence, it can be helpful to consider the insights of key experts in the field. A lot is still unknown, and although this Delphi consensus sheds some light on missing information, further research is needed to fill in the blanks in our knowledge regarding gray areas of evidence, as detailed below.

### Preoperative Considerations

Consensus was achieved on various preoperative aspects, such as the use of CTA with dedicated postprocessing software for sizing and planning, which is in line with literature and the European Society for Vascular Surgery (ESVS) and Society of Vascular Surgery (SVS) guidelines.^9,15,16^ It would be optimal, though, to have a structured protocol with consideration of anatomical factors, such as tortuosity, calcification, and thrombus, to highlight a unified approach to planning to prevent complications in challenging iliac landing zones.^17,18^ In the current ESVS guidelines no clear cut-off values for safe landing zones are given in the recommendations. Instead the recommendation is that EVAR outside the IFU’s is not recommended in elective setting, something that is supported with low level evidence.^9^ In this Delphi consensus a diameter of >24 mm has been defined as hostile, and consensus was reached that the maximum diameter of an ideal landing zone should be <22 mm. This is not mirrored in the current IFUs included in the ESVS guidelines, which give a range of 18 to 25 mm as a maximum diameter of the common iliac artery.^9^ Though these diameters are found to be acceptable in the IFU, the experts believe there is a significant risk in exceeding 22 mm diameter. This is in line with literature showing that landing in common iliac arteries with a diameter >22 mm is prone to type IB endoleaks.^19,20^ The IFUs also consider a length of 10 to 20 mm as a safe landing zone whereas the consensus by the experts was that <15 mm is considered a hostile landing zone.^9^ The guidelines suggest an iliac fixation length of 20 mm, with some evidence indicating that a length greater than 20 mm may reduce the risk of proximal stent graft migration.^9,17^ However, this is not an official recommendation due to the lack of high level evidence.

### Intraoperative Management

In the interaoperative phase, a clear preference for angiography to assess the distal seal was shown, which was consistent with the current practices of a large portion of our Delphi panel.

However, it is important to acknowledge that cone-beam CT has gained attention worldwide as an intraoperative imaging modality, particularly for its ability to detect endoleaks and assess the circumferential seal. Some panelists also mentioned the use of intravascular ultrasound (IVUS), a contrast-free alternative occasionally employed for intraoperative imaging.^21^ While angiography remains the predominant method among our panelists, there is growing recognition that cone-beam CT and possibly IVUS offer valuable complementary options, which may eventually play a larger role in intraoperative management when availability increases.

Consensus was reached on immediate intervention for an intraoperative type IB endoleak, even when it is considered low flow. There is, however, no solid evidence that shows whether a low-flow type IB endoleak will or will not resolve without immediate intervention.^19^ Future studies should provide more clarity about the urgency to intervene. In line with the recommendations of the current ESVS guidelines, the preferred method of treating a IB endoleak during the index procedure depends on the landing zone, where elongation without stenting over the internal iliac artery is preferred when the landing zone is suitable.^9^ There is a cautionary note against the bell-bottom technique, in line with literature showing flared iliac limbs increase the risk of a type IB endoleak.^22^

### Postoperative Follow-Up

Postoperatively, the emphasis on CTA for follow-up imaging and criteria for evaluating distal seal length set a standard for long-term assessments. In the current ESVS guidelines, it is advised that iliac-related factors including sealing length should be included in the follow-up strategy.^9^ The panelists agreed that changes in the distal landing zone are best viewed using CTA and should be compared with baseline findings during follow-up. This is supported by recent literature that shows how the iliac seal may diminish over time due to graft displacement and progression of disease in the iliac arteries, making it important to evaluate the sealing zone in detail, preferably using CTA.^19,23^ Exact evaluation of the distal sealing zone and modifications over time on cross-sectional CTA may be difficult to objectify. This opens the room to implementation of more accurate post processing analytic methods. Although follow-up schedules vary greatly in different centers, the panelists did agree that the first CTA should be done within two months and the second within the first year.^24^ This is more in line with the guidelines of the SVS and the American Heart Association that advise obtaining a CT after 1 year, in contrast to the ESVS guidelines, which advise obtaining a CT within 5 years if the patient is deemed at low risk for complications.^7–9^

Consensus was also reached on how to best deal with a diminishing distal seal without clear type IB endoleaks. The guidelines advise treating type IB endoleaks promptly; however, they do not offer advice on how to manage a diminishing seal, merely that compromised sealing zones may be considered for intervention.^7,9^ The expert panel agreed that follow-up and imaging should be intensified when the seal diminishes. There was no consensus on when and how to intervene when the seal diminishes, something that is also lacking in the current guidelines. The implementation of intensified surveillance and how to manage this was not investigated in this study, but should be a topic for further research to improve tailored care for EVAR patients to be able to make recommendations at what stage an intervention is deemed most appropriate.

Overall, there is added value in structuralizing pre- and postoperative imaging and assessing the iliac landing zone. This Delphi consensus provides a basis for future guidelines and shows where solid evidence is lacking concerning the iliac landing zone. Level 1 evidence, consisting of randomized controlled trials, is challenging in this field. Larger retro- and prospective multicentre registries studies investigating the iliac landing zone and type IB endoleaks are needed to increase the knowledge. Until these studies are conducted, the advice given by the expert panel represents a practical source of reference for practicing physicians. A Delphi consensus approach highlights existing knowledge gaps rather than providing definitive conclusions. We emphasize that our findings are intended to guide future research and discussions, serving as a catalyst for the development of more robust, evidence-based guidelines as further empirical data becomes available.

### Study Limitations

The findings from this study must be interpreted within the context of its limitations. First, the Delphi methodology may be subject to intrinsic shortcomings. Delphi studies have been criticized because the researchers choose the included items, thereby potentially introducing bias. To counteract this, the expert panel had the opportunity to modify and comment on statements or suggest additional ones. Second, the inclusion criteria for the experts meant a random selection was not feasible; thus, a large, preselected group of international experts proposed by the core team was invited, potentially introducing selection bias because they might not fully represent actual worldwide expertise. Because only 77 of our intended 127 panelist responded to all rounds, there was more homogeneity in our panel despite our efforts to create a diverse panel. Even though the non-response rate between the rounds was low, the initial refusal of participation in the first round might cause a selection bias.

Our primary focus was endoleaks and the failure of seal; hence, there is an absence of statements regarding iliac limb occlusion and patency, which represent an additional clinical concern. Although some statements did not reach consensus and were rejected from the final formulation, this may not be equivalent to the assumption they would not address clinically relevant questions. Therefore, consensus statements should only be considered as evidence in progress to be further investigated and confirmed by clinical studies, if possible, and need to be implemented in daily practice with proper clinical judgment. To mitigate this limitation, clinical practice guidelines from recognized scientific societies were consulted when available to ensure proposed statements were in agreement whenever possible. In addition, the statements concerning calcifications, thrombus and tortuosity were generic and did not address the different methods for quantifying these morphological parameters. Future studies may dive further into the grading of these parameters and their influence on sealing durability.

## Conclusion

This international expert-based Delphi consensus establishes a comprehensive set of consensus-driven recommendations focused on the definition and management of hostile iliac landing zones in EVAR. The key recommendation of this study is the novel definition of a hostile iliac landing zone as short (< 15 mm), wide (>24 mm), or conical (>10% diameter difference along the landing zone). Although consensus was achieved on several critical aspects, the study also reveals ongoing debates and considerations that warrant further exploration, including how to tackle diminishing seal without a type IB endoleak.

## Supplemental Material

sj-docx-1-jet-10.1177_15266028241295919 – Supplemental material for Transatlantic Delphi Consensus on the Common Iliac Artery Sealing Zone in Endovascular Aorto-Iliac Aneurysm Repair (the DECIDE Study) Supplemental material, sj-docx-1-jet-10.1177_15266028241295919 for Transatlantic Delphi Consensus on the Common Iliac Artery Sealing Zone in Endovascular Aorto-Iliac Aneurysm Repair (the DECIDE Study) by Maria-Annette Kooijman, Mario D’Oria, Luca Bertoglio, Isabelle Van Herzeele, Ross Milner, Jean-Paul P.M. de Vries and Richte C.L. Schuurmann in Journal of Endovascular Therapy
