# Supplementary material for: Transatlantic Delphi Consensus on the Common Iliac Artery Sealing Zone in Endovascular Aorto-Iliac Aneurysm Repair (the DECIDE Study)
Source: J Endovasc Ther. 2024 Nov 20;33(3):1537–47. doi: 10.1177/15266028241295919 (PMC13176816; doi:10.1177/15266028241295919)
Supplement: sj-docx-1-jet-10.1177_15266028241295919 – Supplemental material for Transatlantic Delphi Consensus on the Common Iliac Artery Sealing Zone in Endovascular Aorto-Iliac Aneurysm Repair (the DECIDE Study) [file sj-docx-1-jet-10.1177_15266028241295919.docx]

Supplementary table S1. Modified statements

| Statement | After round 1 | After round 2 | R2 |
| --- | --- | --- | --- |
| 1 | Modified and resubmitted in round 2 | Unmodified resubmitted in round 3 | 1 |
| 2 | Modified and resubmitted in round 2 | Unmodified resubmitted in round 3 | 2 |
| 3 | Modified and resubmitted in round 2 | Unmodified resubmitted in round 3 | 3 |
| 4 | Modified and resubmitted in round 2 | Unmodified resubmitted in round 3 | 4 |
| 5 | Not present in round 1, added in round 2 after feedback panel | Unmodified resubmitted in round 3 | 5 |
| 6 | Modified and resubmitted in round 2 | Modified and resubmitted in round 3 | 6 |
| 7 | Modified and resubmitted in round 2 | Unmodified resubmitted in round 3 | 7 |
| 8 | Modified and resubmitted in round 2 | Unmodified resubmitted in round 3 | 8 |
| 9 | Not present in round 1, added in round 2 after feedback panel | Unmodified resubmitted in round 3 | 9 |
| 10 | Modified and resubmitted in round 2 | Unmodified resubmitted in round 3 | 10 |
| 11 | Modified and resubmitted in round 2 | Unmodified resubmitted in round 3 | 11 |
| 12 | Not present in round 1, added in round 2 after feedback panel | Unmodified resubmitted in round 3 | 12 |
| 13 | Not present in round 1, added in round 2 after feedback panel | Modified and resubmitted in round 3 | 13 |
| 14 | Modified and resubmitted in round 2 | Unmodified resubmitted in round 3 | 14 |
| 15 | Not present in round 1, added in round 2 after feedback panel | Dropped | 15 |
| 16 | Not present in round 1, added in round 2 after feedback panel | Unmodified resubmitted in round 3 | 16 |
| 17 | Not present in round 1, added in round 2 after feedback panel | Unmodified resubmitted in round 3 | 17 |
| 18 | Dropped | - | - |
| 19 | Modified and resubmitted in round 2 | Unmodified resubmitted in round 3 | 18 |
| 20 | Not present in round 1, added in round 2 after feedback panel | Unmodified resubmitted in round 3 | 19 |
| 21 | Modified and resubmitted in round 2 as 2 separate statements | Unmodified resubmitted in round 3 | 20 |
| 22 |  | Unmodified resubmitted in round 3 | 21 |
| 23 | Modified and resubmitted in round 2 as 3 separate statements | Unmodified resubmitted in round 3 | 22 |
| 24 |  | Unmodified resubmitted in round 3 | 23 |
| 25 |  | Unmodified resubmitted in round 3 | 24 |
| 26 | Not present in round 1, added in round 2 after feedback panel | Unmodified resubmitted in round 3 | 25 |
| 27 | Merged with 28 and resubmitted in round 2 | Modified and resubmitted in round 3 | 26 |
| 28 | Merged with 27 and resubmitted in round 2 |  | 26 |
| 29 | Modified and resubmitted in round 2 | Unmodified resubmitted in round 3 | 27 |
| 30 | Modified and resubmitted in round 2 | Unmodified resubmitted in round 3 | 28 |
| 31 | Modified and resubmitted in round 2 | Unmodified resubmitted in round 3 | 29 |
| 32 | Modified and resubmitted in round 2 | Unmodified resubmitted in round 3 | 30 |
| 33 | Modified and resubmitted in round 2 | Unmodified resubmitted in round 3 | 31 |
| 34 | Modified and resubmitted in round 2 | Unmodified resubmitted in round 3 | 32 |
| 35 | Modified and resubmitted in round 2 | Unmodified resubmitted in round 3 | 33 |
| 36 | Modified and resubmitted in round 2 as 3 separate statements | Unmodified resubmitted in round 3 | 34 |
| 37 |  | Dropped | 35 |
| 38 |  | Unmodified resubmitted in round 3 | 36 |
| 39 | Modified and resubmitted in round 2 | Unmodified resubmitted in round 3 | 37 |
